# Campylobacter sputorum subsp. bovis subsp. nov., isolated from cattle, and an emended description of Campylobacter sputorum

**DOI:** 10.1099/ijsem.0.006571

**Published:** 2024-11-13

**Authors:** William G. Miller, Tina G. Williams, Delilah F. Wood, Mary H. Chapman

**Affiliations:** 1 Produce Safety and Microbiology Research Unit, Agricultural Research Service, U.S. Department of Agriculture, Albany, CA, USA; 2 Bioproducts Research Unit, Agricultural Research Service, U.S. Department of Agriculture, Albany, CA, USA

**Keywords:** California, *Campylobacter sputorum*, cattle, novel subspecies

## Abstract

Six urease-negative *Campylobacter* strains were isolated from cattle faeces over a 19-month period from 2009 to 2010. These strains were initially identified as *Campylobacter sputorum* by 16S rRNA gene and *atpA* typing. Initial studies characterizing these strains by multilocus sequence typing and genome sequencing further supported their classification as *C. sputorum* but indicated that these strains form a divergent clade within the species. A polyphasic study was undertaken here to clarify their taxonomic position. Phylogenetic analyses were performed based on 16S rRNA gene sequences and the concatenated sequences of 330 core genes, with the latter analysis also placing the six strains into a clade distinct from the three *C. sputorum* biovars. Pairwise digital DNA–DNA hybridization values identified these strains as *C. sputorum*, and the pairwise average nucleotide identity values were consistent with those observed between current *Campylobacter* subspecies pairs. Standard phenotypic testing was also performed. All strains are microaerobic, anaerobic, motile, Gram-negative and oxidase- and catalase-positive; cells are curved rods or spirals. Strains can be distinguished from the *C. sputorum* biovars by the presence of alkaline phosphatase activity and triphenyltetrazolium chloride reduction and absence of nitrate reduction. The data presented here show that these strains represent a novel subspecies within *C. sputorum*, for which the name *C. sputorum* subsp. *bovis* subsp. nov. (type strain RM8705^T^=LMG 32300^T^=CCUG 75470^T^) is proposed.

## Data Summary

One supplementary figure and four supplementary tables are provided in the online version of this article. All supplementary data are available through Figshare at https://doi.org/10.6084/m9.figshare.27105994.

## Introduction

*Campylobacter sputorum* is a non-thermotolerant *Campylobacter* that has been recovered from cattle [1,2], sheep [3,4], swine [5] and dogs [6]. Although strains of this species have been isolated from humans [2,7, 8], its pathogenicity is presently undetermined. The taxonomic evolution of *C. sputorum* is long and complex. Originally isolated in 1914 from a human patient with bronchitis [9], it was named ‘*Vibrio sputorum*’ by Prévot [10] and subsequently characterized as a catalase-negative anaerobe [11]. The related species ‘*Vibrio bubulus*’ [12] and ‘*Vibrio faecalis*’ [3] were also described, with the latter being distinguishable from the other two by the presence of catalase activity. Loesche *et al*. [13] demonstrated that ‘*V. sputorum*’ and ‘*V. bubulus*’ were microaerophiles and proposed that these two taxa were subspecies of the same species; ‘*V. sputorum* var. *sputorum*‘ and ‘*V. sputorum* var. *bubulus*’ could be differentiated based on tolerance to both 3.5% (w/v) NaCl and 1% (w/v) bile. These two taxa were assigned to the genus *Campylobacter* by Véron and Chatelain in 1973 [14] and a third *C. sputorum* subspecies, *C. sputorum* subsp. *mucosalis*, was proposed by Lawson *et al*. [15,16]. *C. sputorum* was reorganized in 1985 by Roop *et al*. [17,18], who demonstrated that subspp. *sputorum* and *bubulus* could not be distinguished by DNA homology and that subsp. *mucosalis* had no significant DNA homology to the other two *C. sputorum* subspecies. This created the novel species *Campylobacter mucosalis* and reclassified the remaining two *C. sputorum* subspecies as *C. sputorum* biovars. ‘*V. faecalis*’ (later ‘*Campylobacter faecalis*’) was added as a third *C. sputorum* biovar [17]. The current taxonomic composition of *C. sputorum* was proposed in 1998 by On *et al*. [2], who determined that the salt and bile tolerance tests used to distinguish bvs. sputorum and bubulus were unreliable. These two biovars were combined into bv. sputorum, and a third biovar, the catalase-negative, urease-positive *C. sputorum* bv. paraureolyticus, was added. The three *C. sputorum* biovars (i.e., bvs. sputorum, faecalis and paraureolyticus) could be readily distinguished based on catalase and urease activities [2].

Six *Campylobacter* strains were recovered from cattle faeces in Monterey County, California, from March 2009 to October 2010 (Table S1, available in the online Supplementary Material). Initially known as *C. sputorum* by 16S rRNA gene sequencing [19], these strains were catalase-positive and urease-negative, the phenotype associated with *C. sputorum* bv. faecalis. However, additional characterization using a novel *C. sputorum* multilocus sequence typing (MLST) method demonstrated that the strain set comprised four sequence types that were distinct from those of *C. sputorum* bv. faecalis [19]; phylogenetic analysis also placed these sequence types into a clade well separated from the clade comprising the three *C. sputorum* biovars [19]. Four of the six cattle strains were typed using *atpA* gene sequencing and also shown to compose a clade separate from the three *C. sputorum* biovars but sister to *C. sputorum* bv. paraureolyticus, thus confirming the MLST results [20]. A complete, gap-free genome of a representative cattle-associated strain, RM8705^T^, was constructed and compared to the complete genomes of *C. sputorum* bv. sputorum RM3237, *C. sputorum* bv. faecalis LMG 8532 and *C. sputorum* bv. paraureolyticus LMG 17589 [21]. The four genomes are relatively colinear, with a high percentage of shared gene content. Nevertheless, while average nucleotide identity (ANI) values ranged between 98 and 99% among the three established biovars, pairwise values between these biovars and strain RM8705^T^ were 94–95% [21]. Additionally, digital DNA–DNA hybridization (dDDH) values were 92–95% between the three biovars but 82–87% when these biovars were compared to strain RM8705^T^ [21]. These dDDH values were all above the 70% threshold recommended to define novel species [22]. However, the ANI values were slightly lower than the proposed 95% value recommended for species delineation [23,24] but similar to ANI values observed between other *Campylobacter* subspecies pairs. This suggested that strain RM8705^T^ and perhaps the other five cattle-associated *C. sputorum* strains were members of a novel *C. sputorum* subspecies [21]. We present here a polyphasic study that provides further evidence that the six cattle-associated strains represent a novel *C. sputorum* subspecies.

## Genomic analysis and taxonomic placement

The five remaining cattle-associated *C. sputorum* strains were sequenced here to draft level using Illumina MiSeq sequencing, as described by Miller *et al.* [25]. The genome metrics are presented in Table S1. Proteins predicted to be encoded by the draft genomes were identified by GeneMark [26]. The draft proteomes were compared to the proteomes of *C. sputorum* bv. sputorum LMG 7795^T^, *C. sputorum* bv. sputorum RM3237, *C. sputorum* bv. faecalis LMG 8532, *C. sputorum* bv. paraureolyticus LMG 17589 and *C. sputorum* strain RM8705^T^ by pairwise BLASTP analysis. A high conservation in gene content was observed among the six cattle-associated strains: 96.8% of the genes identified in strain RM8705^T^ were also identified in each of the other five strains. Additionally, loci described as absent in strain RM8705^T^ [21], when strain RM8705^T^ was compared to the *C. sputorum* bv. strains, are also missing from the other five cattle-associated strains. These include a *ttrACBSR* tetrathionate reductase locus, the *ceuBCDE-exbBD-tonB* ferric enterobactin transporter locus and a 15-gene locus putatively encoding an allophanate hydrolase, radical S-adenosyl methionine-associated transferases, membrane proteins and rhodanese and Fido/AMPylation domain-containing proteins. As expected, all six genomes contain the *katA* catalase gene and do not encode any urease locus proteins. The 16S rRNA genes of these six strains are 1742 bp, which are longer than most 16S rRNA genes typically observed in *Campylobacter* but consistent with those reported previously in *C. sputorum* [2,27, 28]. These larger 16S rRNA genes are the result of structured intervening sequences (IVSs) in helix 10 of the 5′ major domain. Such IVSs are also found at the same position within the 16S rRNA genes of some strains in other *Campylobacter* species, e.g., *Campylobacter curvus* and *Campylobacter helveticus* [29,30].

**Fig. 1.**
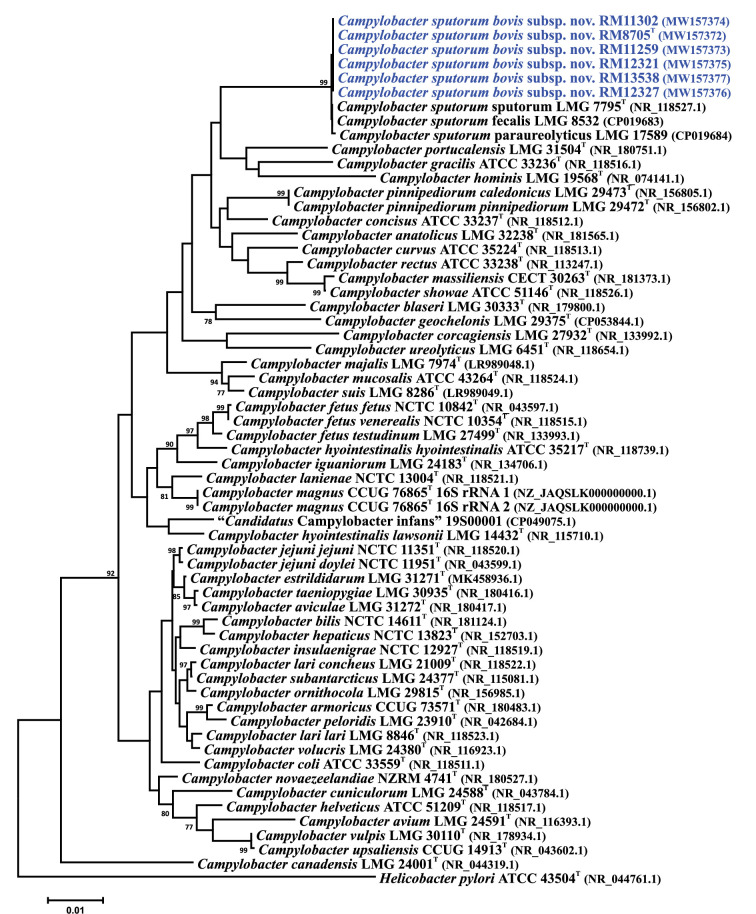
16S rRNA gene phylogenetic tree showing the position of *C. sputorum* subsp. *bovis* subsp. nov. within the genus *Campylobacter*. Included in the tree are the 16S rRNA gene sequences of the six subsp. *bovis* strains and *Campylobacter* type strain 16S rRNA gene sequences. Bootstrap values of ≥75%, generated from 1000 replicates, are shown at the nodes. GenBank accession numbers (in parentheses) are provided for each strain. The *Campylobacter magnus* type strain contains two different 16S rRNA gene sequences and both are included in the tree. The *Helicobacter pylori* type strain was used to root the tree. The scale bar represents nucleotide sequence divergence.

**Fig. 2.**
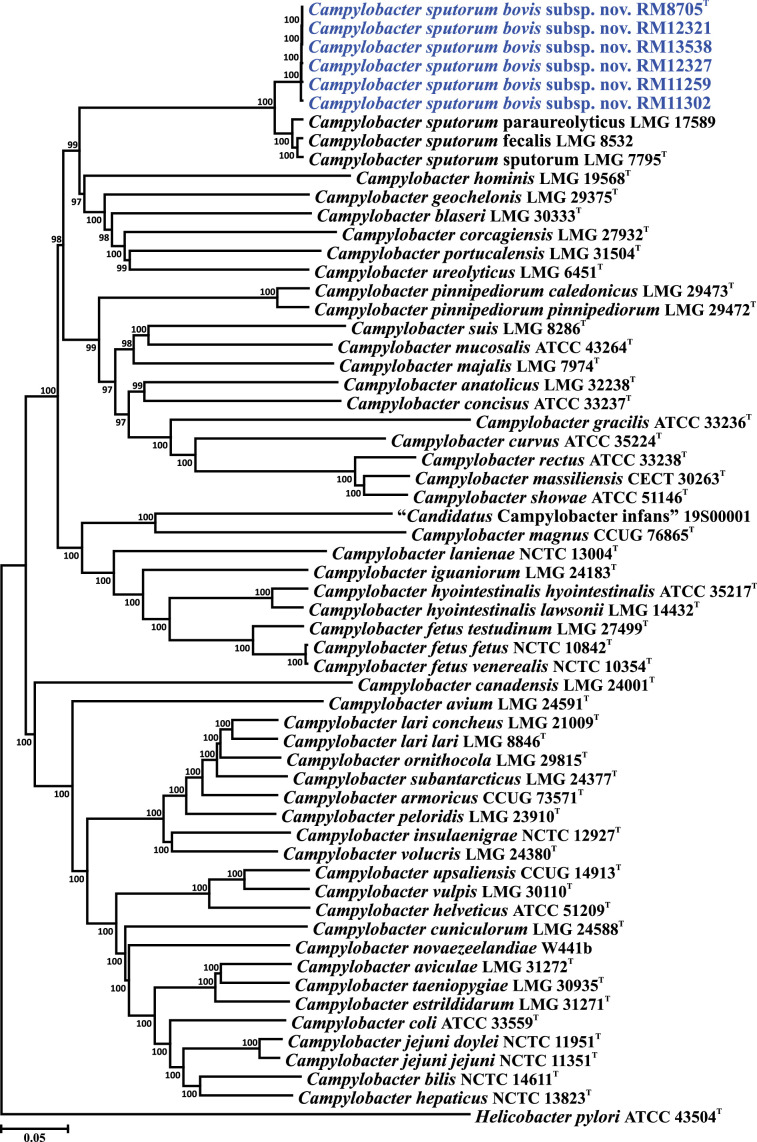
Core genome phylogeny of *C. sputorum* subsp. *bovis* subsp. nov. within the genus *Campylobacter*. Phylogeny is based on the concatenated nucleotide sequence alignment of 330 core genes and compares the six subsp. *bovis* strains, the three *C. sputorum* subsp. *sputorum* subsp. nov. biovars and *Campylobacter* type strains. Bootstrap values of >75%, generated from 1000 replicates, are shown at the nodes. The *Helicobacter pylori* type strain was used to root the tree. The scale bar represents nucleotide sequence divergence.

16S rRNA gene and core gene sequence phylogenetic analyses were performed using the six cattle-associated *C. sputorum* strains and the full set of *Campylobacter* type strains. 16S rRNA genes were aligned using Clustal X. The nucleotide sequences of 330 core genes were extracted from the *C. sputorum* and *Campylobacter* type strain genomes and aligned individually using muscle [31] in Geneious (ver. 2024.0.5); within Geneious, the 330 core gene alignments were concatenated alphabetically by gene name into a single alignment. The core genes used and their cognate locus tags are listed in Table S2. Phylogenetic trees were constructed within mega version 6.06 [32] using the neighbour-joining method [33], the Kimura 2-parameter distance estimation method [34] and 1000 bootstrap replicates. The 16S rRNA gene sequences of the cattle-associated *C. sputorum* strains are nearly identical (~98% similarity; data not shown) to those of the three *C. sputorum* biovars (Fig. 1). With the exception of one single-nucleotide polymorphism (SNP), the observed differences between the *C. sputorum* 16S rRNA gene sequences reside in the IVSs. Analysis of the *C. sputorum* IVSs shows that those from the six cattle-associated strains are identical and that multiple SNPs can be observed when these IVSs are compared to those of the *C. sputorum* biovars (Fig. S1A). These sequence differences are also reflected in the predicted two-dimensional structures of the IVSs (Fig. S1B–D). Therefore, although the *C. sputorum* 16S rRNA genes are nearly indistinguishable phylogenetically, they can be sorted into two discrete groups on the basis of the sequence and structure of the 16S rRNA gene internal spacer. Core gene phylogeny demonstrates that the six novel strains can be clearly distinguished from the other *Campylobacter* species and form a discrete sister clade to the three *C. sputorum* biovars (Fig. 2). These data are consistent with previous studies using MLST or *atpA* typing, in which these strains are also composed of clades similar to but distinct from *C. sputorum*.

These phylogenetic analyses further indicated that the six cattle-associated strains are either *C. sputorum* or highly related to *C. sputorum*. To clarify the taxonomic position of the six strains, ANI [23] and dDDH [35] analyses were performed. A dDDH value of 70% for species delineation is approximately equivalent to an ANI value of 95% [23,24]. dDDH analyses were performed using the Genome-to-Genome Distance Calculator (GGDC ver. 3.0; https://ggdc.dsmz.de/ggdc.php# [36,37]); GGDC formula 3 was used for all calculations, as recommended by the latest minimal standards for *Campylobacter* [38]. Pairwise dDDH values between each of the six strains and the *C. sputorum* biovars are >70% (81.2–86.7%; Table 1), indicating the placement of all six cattle strains within *C. sputorum*. ANI analyses were performed here using JSpecies (ver. 1.2.1) [24]. Pairwise ANI values between the six strains and the three *C. sputorum* biovars ranged from 94.5 to 95.5% (Table 1), which is either slightly below or slightly above the species delineation threshold. However, within *Campylobacter*, pairwise ANI values between the six established subspecies pairs range from 91.9 to 96.1% (Table S3A). The pairwise ANI values between the six strains and the three *C. sputorum* biovars fall within this range. Moreover, the relatively low dDDH values described above are also consistent with the dDDH values observed between current *Campylobacter* subspecies (Table S3A).

**Table 1.** Pairwise dDDH and ANI values between *C. sputorum* subsp. *bovis* subsp. nov. and related *Campylobacter* taxa

dDDH	RM8705^T^		RM11259		RM11302		RM12321		RM12327		RM13538	
*C. sputorum* bv. paraureolyticus LMG 17589	86.7		86.6		85.2		86.5		85.6		86.4	
*C. sputorum* bv. faecalis LMG 8532	85.2		85.3		83.9		85.1		84.7		85.1	
*C. sputorum* bv. sputorum ATCC 35980^T^	82.4		83.4		81.2		82.5		82.2		82.4	
*C. ureolyticus* LMG 6451^T^	14.6		14.4		14.5		14.4		14.5		14.5	
*C. blaseri* LMG 30333^T^	14.3		14.2		14.2		14.2		14.3		14.2	
*C. corcagiensis* LMG 27932^T^	14.3		14.3		14.2		14.2		14.3		14.2	
*C. portucalensis* LMG 31504^T^	14.3		14.4		14.2		14.3		14.3		14.3	
**ANI**	**1**	**2**	**3**	**4**	**5**	**6**	**7**	**8**	**9**	**10**	**11**	**12**
*C. sputorum bovis* RM8705^T^	–	100	100	99.8	99.8	99.7	94.6	95.4	94.9	70.6	70.3	70.3
*C. sputorum bovis* RM12321	100	–	100	99.8	99.8	99.7	94.6	95.4	95.0	70.8	70.3	70.3
*C. sputorum bovis* RM13538	100	100	–	99.9	99.8	99.7	94.7	95.5	95.0	70.7	70.4	70.2
*C. sputorum bovis* RM12327	99.8	99.8	99.9	–	99.8	99.8	94.6	95.3	94.9	70.9	70.3	70.2
*C. sputorum bovis* RM11259	99.8	99.8	99.8	99.8	–	99.7	94.7	95.3	95.0	70.9	70.3	70.3
*C. sputorum bovis* RM11302	99.7	99.7	99.7	99.8	99.7	–	94.6	95.3	95.0	70.7	70.3	70.1
*C. sputorum* sputorum ATCC 35980^T^	94.5	94.6	94.5	94.5	94.6	94.5	–	97.8	98.6	71.2	70.4	70.4
*C. sputorum* paraureolyticus LMG 17589	95.3	95.3	95.3	95.3	95.3	95.3	97.9	–	97.9	70.6	70.4	70.2
*C. sputorum* faecalis LMG 8532	94.7	94.8	94.8	94.9	94.8	94.8	98.6	97.9	–	70.7	70.5	70.3
*C. portucalensis* LMG 31504^T^	70.7	70.7	70.7	70.9	70.9	70.7	70.9	70.4	70.4	–	73.3	71.7
*C. ureolyticus* LMG 6451^T^	70.5	70.5	70.4	70.3	70.5	70.5	70.4	70.5	70.5	73.6	–	72.1
*C. blaseri* LMG 30333^T^	70.4	70.4	70.4	70.3	70.4	70.4	70.4	70.3	70.4	72.0	72.1	–

Pairwise dDDH values between the *C. sputorum* subsp. *bovis* type strain and other *Campylobacter* taxa >14% and pairwise ANI values between *C. sputorum* subsp. *bovis* and other *Campylobacter* taxa >70% are shown.

Therefore, these results suggest that the six strains recovered from cattle form a novel *C. sputorum* subspecies, for which we propose the name *C. sputorum* subsp. *bovis* subsp. nov. The creation of this subspecies would move the current *C. sputorum* bv. strains into the novel subspecies *C. sputorum* subsp. *sputorum* subsp. nov., with *C. sputorum* subsp. *sputorum* bv. sputorum strain LMG 7795^T^ as the type strain of the subspecies. While not a requirement for defining novel subspecies, it is also noteworthy that there is a consistent difference in the G+C content between the two proposed subspecies, with subsp. *bovis* values ranging between 29.19 and 29.26 mol% and subsp. *sputorum* values ranging between 29.63 and 29.72 mol% (Table S3B). Although these G+C content data may change with the addition of new genomic data, it provides further supporting evidence of the taxonomic separation of the two proposed subspecies.

## Morphology and phenotypic characterization

**Fig. 3.**
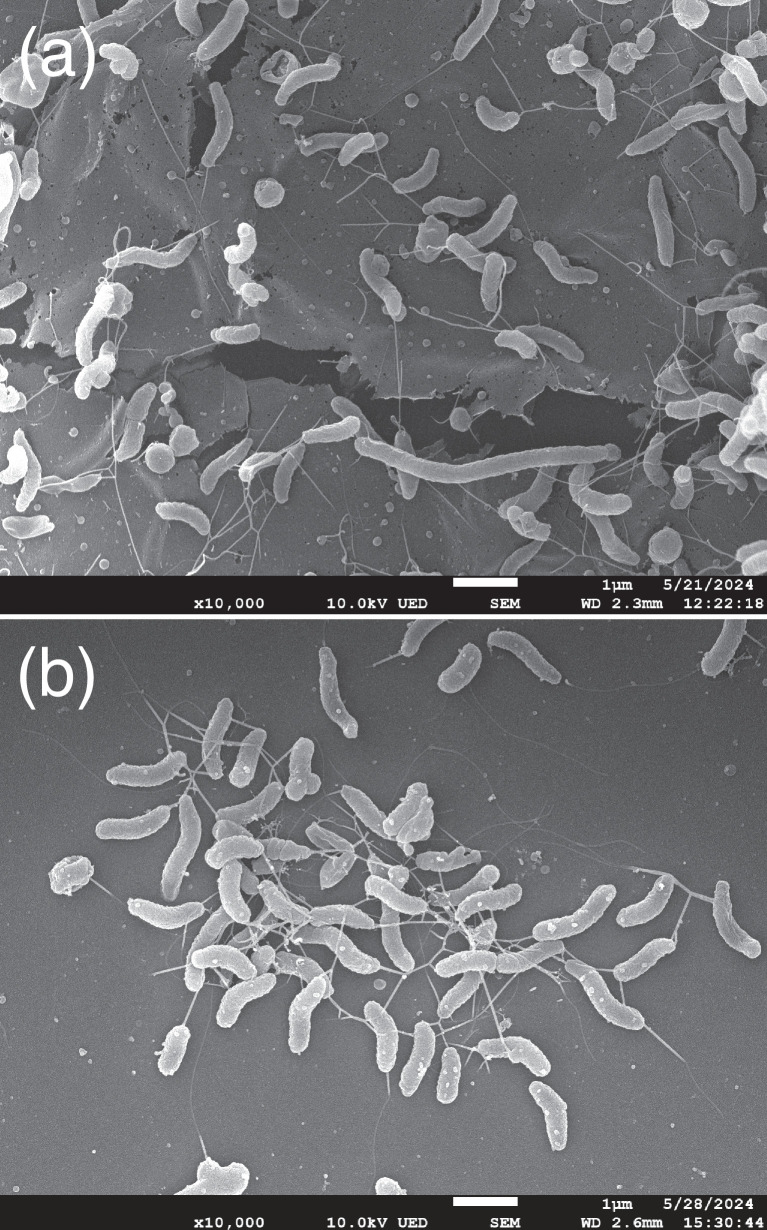
Scanning electron micrograph images of *C. sputorum* subsp. *bovis* subsp. nov. strains (a) RM8705^T^ (=LMG 32300^T^) and (b) RM11302 (=LMG 32301) at ×10 000 magnification. Sample preparation and microscopy were performed as described by Miller *et al*. [39].

Cells from these strains are motile with a cellular morphology typical of other *Campylobacter* species, i.e., a mixture of curved rods and spiral cells (Fig. 3). The six *C. sputorum* subsp. *bovis* subsp. nov. strains were characterized using the standard phenotypic tests defined in the minimal standards for *Campylobacter* [38]. Strains were tested for: growth at 30, 37 and 42 °C under microaerobic conditions; growth at 37 °C under aerobic and anaerobic conditions; motility; selenite, indoxyl acetate and triphenyltetrazolium chloride (TTC) reduction; oxidase, catalase, alkaline phosphatase, hippuricase, urease and nitrate reductase activity; growth on modified charcoal–cefoperazone–deoxycholate agar (mCCDA) plates or media amended with 2% (w/v) NaCl, 1% (w/v) glycine or 0.04% (w/v) TTC; α-haemolysis on anaerobe basal agar (ABA; Oxoid) amended with 5% lysed horse blood (Innovative Research, Novi, MI) [ABA with blood (ABA-B)]; H_2_S production on triple sugar iron (TSI) agar; and resistance to 30 mg l^−1^ nalidixic acid or 30 mg l^−1^ cephalothin. All tests were performed in triplicate using appropriate positive and negative controls [39]. The results of these phenotypic tests are presented in Table 2. All strains: could grow under microaerobic conditions at 37 and 42 °C or under anaerobic conditions at 37 °C, but not aerobically or at 30 °C microaerobically; were motile; were oxidase-, catalase- and alkaline phosphatase-positive and urease- and hippuricase-negative; did not reduce nitrate but could reduce both selenite and TTC; produced H_2_S on TSI agar; did not hydrolyse indoxyl acetate; could grow on media supplemented with 2% (w/v) NaCl or 1% (w/v) glycine and weakly on media supplemented with 0.04% (w/v) TTC and were resistant to both 30 mg l^−1^ nalidixic acid and 30 mg l^−1^ cephalothin.

**Table 2.** Phenotypic characteristics of *C. sputorum* subsp. *bovis* subsp. nov. and related *Campylobacter* species

	1	2	3	4	5	6	7	8	9	10
Motility	+	+	+	+	−	−	−	−	−	−
Temperature (atmosphere)										
37 °C (aerobic)	−	−	−	−	−	−	−	−	−	−
30 °C (microaerobic)	−	M	M	M	+	+	+	−	U	+
37 °C (microaerobic)	+	+	+	+	+	+	+	+	+	+
42 °C (microaerobic)	+	V	V	V	+	+	−	−	+	V
37 °C (anaerobic)	+	+	+	+	+	+	+	+	w	+
Oxidase	+	+	+	+	+	+	+	+	+	+
Catalase	+	−	+	−	+	+	+	−	−	F
Urease	−	−	−	+	+	+	−	−	−	+
Alkaline phosphatase	+	−	−	−	+	+	−	−	U	−
Hippuricase	−	−	−	−	−	−	+	−	−	−
Indoxyl acetate hydrolysis	−	−	−	−	+	V	−	−	−	F
Reduction:										
Nitrate	−	+	M	+	+	M	+	V	−	+
Selenite	+	V	V	V	U	U	−	−	U	−
TTC	+	−	−	−	U	U	−	−	U	−
H_2_S production on TSI	+	+	+	+	+	+	−	−	−	−
α−Haemolysis	−	+	+	+	−	−	−	−	−	V
Growth on:										
2% (w/v) NaCl	+	+	+	+	U	+	+	U	−	+
1% (w/v) glycine	+	+	+	+	w	+	+	+	V	+
0.04% (w/v) TTC	w	−	−	−	U	−	U	−	−	−
mCCDA	V	M	M	M	−	U	+	U	U	V
Resistance to:										
Nalidixic acid (30 mg l^−1^)	R	V	V	V	S	R	R	V	U	S
Cephalothin (30 mg l^−1^)	R	S	S	S	S	S	S	S	U	S

Species: 1, *C. sputorum* subsp. *bovis* subsp. nov. (*n* = 6); 2, *C. sputorum* bv. sputorum; 3, *C. sputorum* bv. faecalis; 4, *C. sputorum* bv. paraureolyticus; 5, *Campylobacter blaseri*; 6, *Campylobacter corcagiensis*; 7, *Campylobacter geochelonis*; 8, *Campylobacter hominis*; 9, *Campylobacter portucalensis* and 10, *Campylobacter ureolyticus*. Positive strains: + (95−100%), M (70−95%), V (30−70%), F (10−30%), − (0−10%); w, weak growth; for antibiotic resistance, S/V/R indicates sensitive, variable or resistant, respectively; U, unknown/data not available. The complete comparison within the genus *Campylobacter* is shown in Table S4. Data for columns 2−10 are derived from the original species descriptions and/or On *et al.* [38].

Strains from both proposed subspecies share multiple phenotypic features that together distinguish them from the other *Campylobacter* taxa (Tables 2 and S4). Phenotypic profiles unique to each subspecies were also observed. As shown here and as described previously [21], the *C. sputorum* subsp. *bovis* strains are uniformly catalase-positive and urease-negative, a phenotype proposed by On *et al*. [2] to classify such strains within *C. sputorum* as bv. faecalis. However, the *C. sputorum* subsp. *bovis* strains do not reduce nitrate and demonstrate alkaline phosphatase activity, reduction of TTC and resistance to cephalothin (Table 2) and are thus clearly distinct from *C. sputorum* subsp. *sputorum*. The absence of nitrate reduction is noteworthy since all six subsp. *bovis* strains contain complete, untruncated nitrate reductase (*napAGHBFLD*) loci. However, alignment of the *C. sputorum* nitrate reductase proteins identified multiple amino acid substitutions within the subsp. *bovis* proteins when they were compared to those of subsp. *sputorum* (data not shown). One or more of these substitutions, as well as possible point mutations in the upstream promoter region, may potentially lead to a loss of nitrate reductase activity. Further work will be necessary to clarify the absence of nitrate reduction in *C. sputorum* subsp. *bovis*.

## Emended description of *Campylobacter sputorum* (Prévot 1940) Véron and Chatelain 1973 (Approved Lists 1980)

The species description is as previously described by On *et al.* [2], with the following emendations. Some strains demonstrate alkaline phosphatase activity, reduce TTC, do not reduce nitrate and grow weakly on blood agar amended with 0.04% TTC. Strains of *C. sputorum* may be assigned to *C. sputorum* subsp. *sputorum* or *C. sputorum* subsp. *bovis* according to their alkaline phosphatase activity and reduction of TTC. *C. sputorum* subsp. *sputorum* strains do not demonstrate alkaline phosphatase activity and do not reduce TTC, while *C. sputorum* subsp. *bovis* strains demonstrate alkaline phosphatase activity and reduce TTC. *C. sputorum* subsp. *sputorum* strains may be further assigned to one of three biovars based on their catalase and urease activities, as previously described [2].

## Description of *Campylobacter sputorum* subsp. *sputorum* subsp. nov.

*Campylobacter sputorum* subsp. *sputorum* (spu.to’rum. L. neut. n. *sputum*, spit, sputum; L. gen. pl. n. *sputorum*, of sputa). Strains conform to the description of *C. sputorum*, as previously described by On *et al.* [2].

## Description of *Campylobacter sputorum* subsp. *bovis* subsp. nov.

*Campylobacter sputorum* subsp. *bovis* (bo’vis. L. gen. n. *bovis*, of a cow, of bovine). Gram-negative cells are motile with a curved or spiral morphology. After 72 h culture at 37 °C under microaerobic conditions on ABA-B, colonies glisten, are opaque, convex and circular with entire margins and are 1–2 mm in diameter. Growth occurs on ABA-B at both 37 and 42 °C under microaerobic conditions and at 37 °C under anaerobic conditions. No growth on ABA-B at 30 °C under microaerobic conditions or at any temperature under aerobic conditions. All strains have oxidase, catalase and alkaline phosphatase activities but no urease activity. Strains do not hydrolyse indoxyl acetate or hippurate or reduce nitrate. All strains reduce selenite and TTC. Produces H_2_S on TSI agar. Growth is supported on ABA-B with a final NaCl concentration of 2% (w/v) and on ABA-B amended with 1% (w/v) glycine. Strains demonstrate weak growth on ABA-B amended with 0.04% TTC. Strains are resistant to 30 mg l^−1^ cephalothin and 30 mg l^−1^ nalidixic acid. Pathogenicity is unknown. Six strains were isolated from cattle. The G+C content of its DNA is 29.2–29.3 mol%. The type strain is RM8705^T^ (=LMG 32300^T^ = CCUG 75470^T^), recovered in 2009 from cow faeces in California. Accession numbers for the 16S rRNA gene and genome sequence of the type strain are MW157372 and CP019685, respectively.

## Supplementary material

10.1099/ijsem.0.006571 Uncited Fig. S1.

10.1099/ijsem.0.006571 Uncited Table S1.

## Abbreviations

ABA: anaerobe basal agar
ABA-B: anaerobe basal agar with blood
ANI: average nucleotide identity
dDDH: digital DNA–DNA hybridization
GGDC: Genome-to-Genome Distance Calculator
IVS: intervening sequence
mCCDA: modified charcoal–cefoperazone–deoxycholate agar
MLST: multilocus sequence typing
TSI: triple sugar iron
TTC: triphenyltetrazolium chloride
