# Supplementary material for: Campylobacter sputorum subsp. bovis subsp. nov., isolated from cattle, and an emended description of Campylobacter sputorum
Source: Int J Syst Evol Microbiol. 2024 Nov 13;74(11):006571. doi: 10.1099/ijsem.0.006571 (PMC12453555; doi:10.1099/ijsem.0.006571)
Supplement: Uncited Fig. S1. [file ijsem-74-06571-s001.pdf]

Fig. S1. Alignments and two-dimensional structures of the *C. sputorum* intervening sequences (IVSs) in helix 10 of the 16S rRNA gene. (A) Alignment of the *C. sputorum* rRNA gene IVSs; alignments were performed using Clustal Omega within Geneious (ver. 2022.0.1). (B-D) Two dimensional IVS structures of (B) *C. sputorum* bvs. sputorum and fecalis (* base present in some bv. sputorum strains; † A in bv. fecalis, G in bv. sputorum), (C) *C. sputorum* bv. paraureolyticus, and (D) *C. sputorum* subsp. *bovis* subsp. nov.; IVS structures were determined using MXfold2 [1].

A

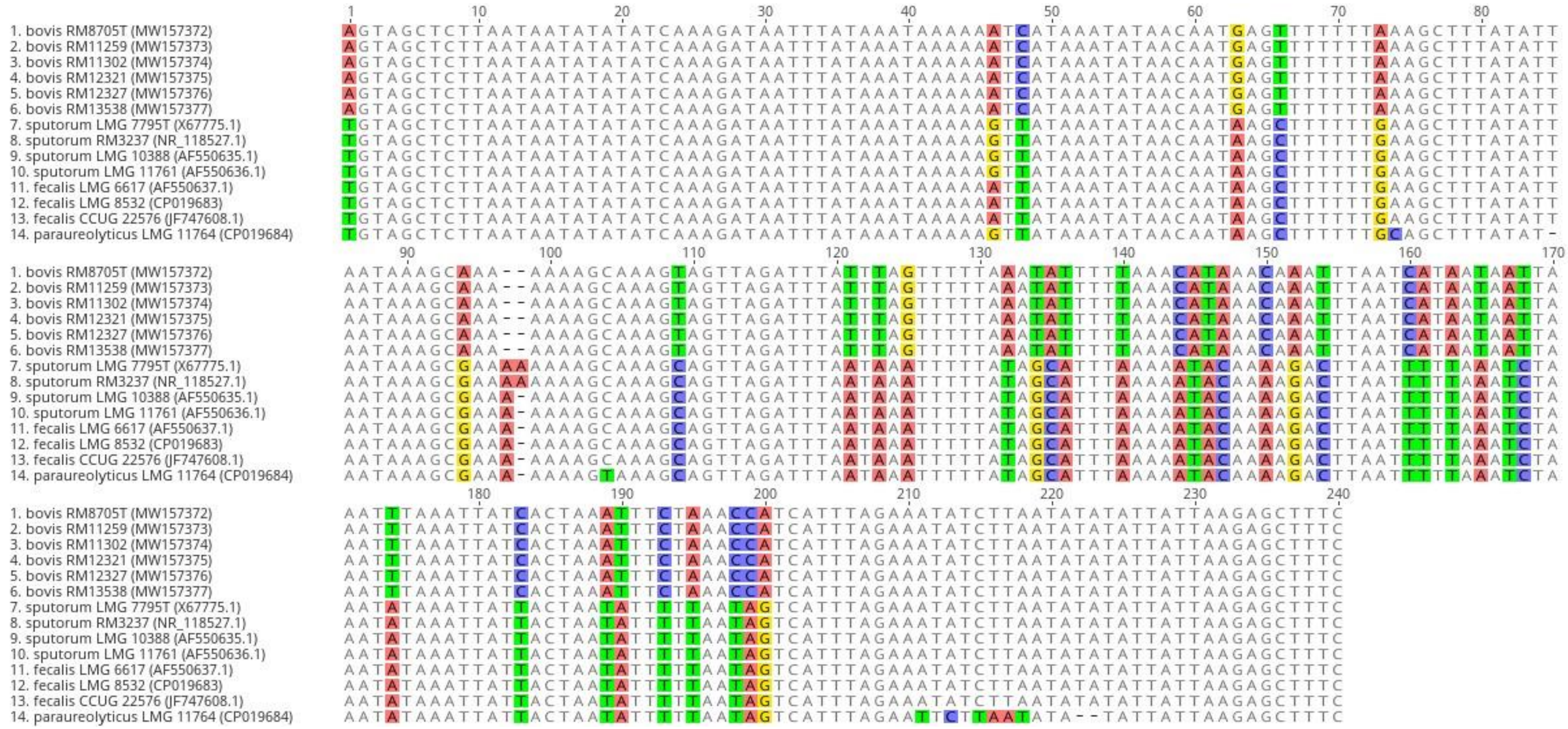

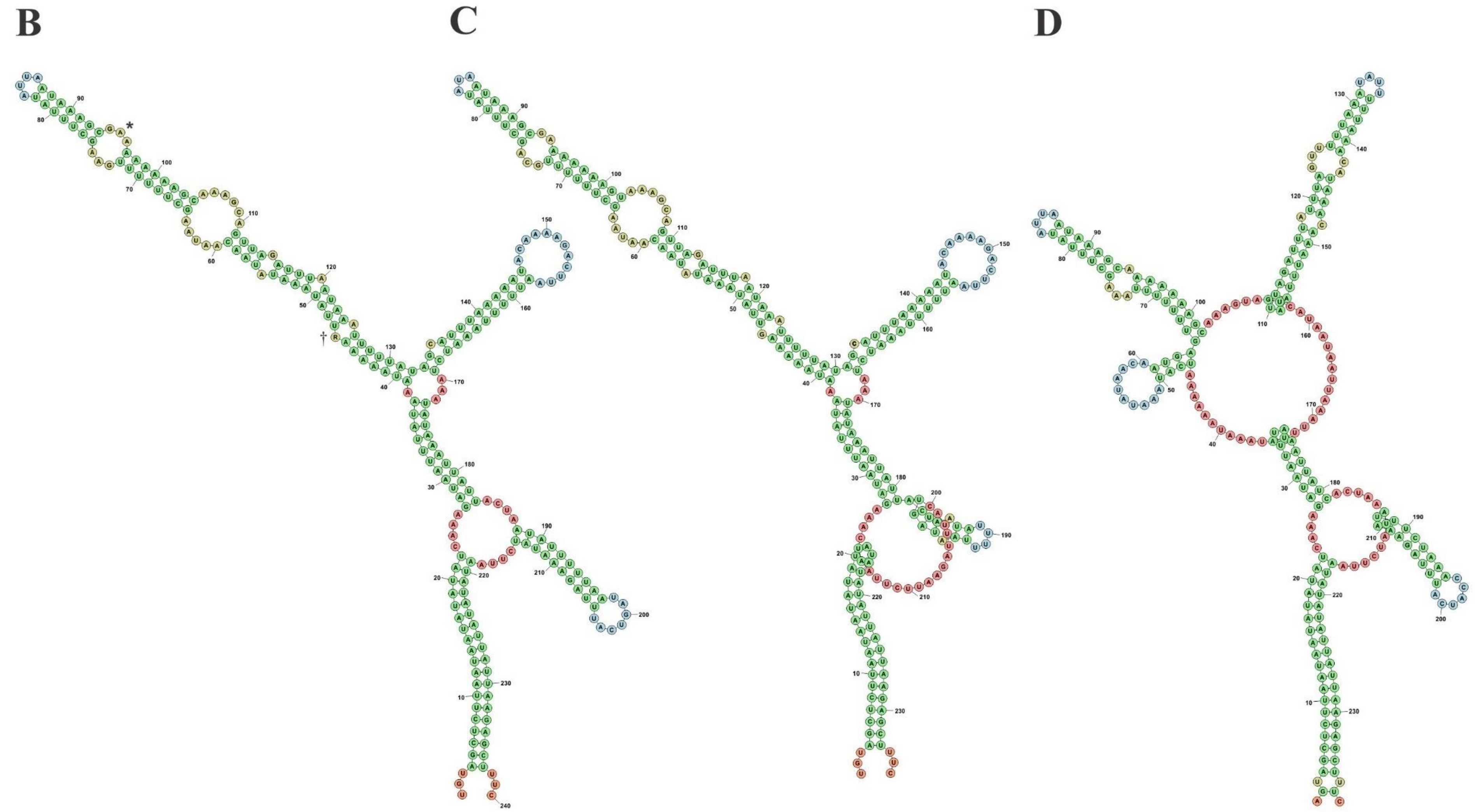

[1]. Sato K, Akiyama M, Sakakibara Y. RNA secondary structure prediction using deep learning with thermodynamic integration. *Nat Commun* 2021;12:941.

Table S1: Strain and genome sequencing data for *C. sputorum* subsp. *bovis* subsp. nov.

Strain	Source	Date	MLST ST	Isolation locale[a]
RM8705[T]	Cow, fecal	18 Mar 2009	ST-8	USA: California: Monterey County [⁑]
RM11259	Cow, fecal	03 Feb 2010	ST-13	USA: California: Monterey County [†]
RM11302	Cow, fecal	18 Feb 2010	ST-14	USA: California: Monterey County [‡]
RM12321	Cow, fecal	13 May 2010	ST-8	USA: California: Monterey County [†]
RM12327	Cow, fecal	13 May 2010	ST-15	USA: California: Monterey County [†]
RM13538	Cow, fecal	28 Oct 2010	ST-8	USA: California: Monterey County [§]

Strain	BioSample	BioProject	Accession # (genome)	Accession # (reads)
RM8705[T]	SAMN03737992	PRJNA284989	CP019685	Not deposited
RM11259	SAMN14886972	PRJNA669413	JADDIO000000000	SRR20751891
RM11302	SAMN14886973	PRJNA669412	JADDIN000000000	SRR20751890
RM12321	SAMN14886974	PRJNA669411	JADDIP000000000	SRR20751906
RM12327	SAMN14886975	PRJNA669410	JADDIQ000000000	SRR20751905
RM13538	SAMN14886976	PRJNA669409	JADDIR000000000	SRR20751900

Strain	Contigs (≥ 5000 bp)	Largest contig	N50	Genome size (Mb)	% G+C	Reads	Bases	Coverage (×)
RM8705[T]	1	N/A	N/A	1.681	29.26	18,699,356	3,311,937,512	1970
RM11259	50 (30)	184,757	93,133	1.646	29.22	1,261,922	311,292,774	189
RM11302	47 (27)	238,580	138,235	1.671	29.19	927,666	227,140,064	136
RM12321	63 (41)	159,778	58,604	1.642	29.21	2,615,426	641,518,491	391
RM12327	56 (30)	162,995	63,119	1.659	29.20	1,039,404	154,492,779	93
RM13538	54 (30)	169,330	89,840	1.643	29.20	1,209,175	177,941,229	108

[a]: identical superscripted symbols indicate identical source locations

Table S3.

(A). ANI and dDDH values among current *Campylobacter* subspecies pairs.

		ANI	dDDH	Model 3 C.I.
C. jejuni jejuni NCTC 11351^T	*C. jejuni doylei* NCTC 11951^T	96.1	68.4	[65.0 - 71.6%]
C. pinnipediorum pinnipediorum LMG 29472^T	*C. pinnipediorum caledonicus* LMG 29473^T	94.3	84.8	[81.6 - 87.5%]
C. hyointestinalis hyointestinalis ATCC 35217^T	*C. hyointestinalis lawsonii* LMG 14432^T	94.3	65.3	[61.9 - 68.5%]
C. lari lari LMG 8846^T	*C. lari concheus* LMG 21009^T	93.1	81.6	[78.3 - 84.6%]
C. fetus fetus NCTC 10842^T	*C. fetus testudinum* LMG 27499^T	91.9	85.3	[82.1 - 88.0%]
C. fetus venerealis NCTC 10354^T	*C. fetus testudinum* LMG 27499^T	92.0	81.0	[77.6 - 84.0%]

(B). %G+C values within *Campylobacter sputorum*.

Strain	G+C mol%
C. sputorum bovis RM8705^T	29.26
C. sputorum bovis RM11259	29.22
C. sputorum bovis RM11302	29.19
C. sputorum bovis RM12321	29.21
C. sputorum bovis RM12327	29.20
C. sputorum bovis RM13538	29.20
C. sputorum sputorum bv. sputorum LMG 7795^T	29.71
C. sputorum sputorum bv. sputorum NCTC 12475	29.71
C. sputorum sputorum bv. sputorum RM3237	29.71
C. sputorum sputorum bv. fecalis LMG 8532	29.72
C. sputorum sputorum bv. paraureolyticus LMG 17589	29.63

Table S4. Phenotypic characteristics of *C. sputorum* subsp. *bovis* subsp. nov. and other *Campylobacter* taxa.

	sput. bovis subsp. nov.	*anatolicus*	*armoricus*	*aviculae*	*avium*	*bilis*	*blaseri*	*canadensis*	*coli*	*concisus*	*corcagiensis*	*cuniculorum*	*curvus*	*estrildidarum*	*fetus fetus*	*fetus venerealis*	*fetus testudinum*	*geochelonis*	*gracilis*	*helveticus*	*hepaticus*	*hominis*	*hyo. hyointestinalis*	*hyo. lawsonii*	*iguaniorum*	*insulaenigrae*
Motility	+	+	+	+	+	+	−	+	+	+	−	+	+	+	+	+	+	−	−	+	+	−	+	+	+	+
Temperature (atmosphere)																										
37 °C (aerobic)	−	−	−	−	−	−	−	−	−	−	−	−	−	−	−	−	F	−	−	−	−	−	−	−	−	−
30 °C (microaerobic)	−	+	−	−	−	U	+	−	−	M	+	U	+	−	+	+	+	+	M	V	U	−	+	+	+	−
37 °C (microaerobic)	+	+	+	+	+	+	+	+	+	+	+	+	+	+	+	+	+	+	+	+	+	+	+	+	+	+
42 °C (microaerobic)	+	+	+	+	+	+	+	+	+	M	+	M	M	+	M	−	M	−	M	+	+	−	+	+	−	−
37 °C (anaerobic)	+	+	+	+	−	−	+	+	−	+	+	−	+	+	V	M	+	+	+	−	−	+	−	+	w	−
Oxidase	+	+	+	+	+	+	+	+	+	V	+	+	+	+	+	+	+	+	−	+	+	+	+	+	+	+
Catalase	+	−	+	−	w	+	+	+	+	−	+	+	−	F	+	+	+	+	V	−	+	−	+	+	+	+
Urease	−	−	+	−	−	−	+	+	−	−	+	−	−	−	−	−	−	−	−	−	−	−	−	−	−	−
Alkaline phosphatase	+	+	−	−	−	U	+	V	−	M	+	−	V	−	−	−	−	−	−	−	U	−	−	F	+	U
Hippuricase	−	−	−	+	+	V	−	+	−	−	−	−	−	M	−	−	−	+	−	−	M	−	−	−	−	−
Indoxyl acetate hydrolysis	−	−	−	−	+	+	+	+	+	−	V	+	V	−	−	−	−	−	M	+	+	−	−	−	−	−
Reduction:																										
Nitrate	−	−	−	V	+	F	+	+	+	F	M	+	+	V	+	M	+	+	M	+	V	V	+	+	+	+
Selenite	+	U	V	+	−	U	U	−	+	F	U	−	−	+	M	F	+	−	−	−	U	−	+	+	−	+
TTC	+	−	V	+	−	U	U	+	+	−	U	V	V	+	−	−	+	−	−	−	U	−	F	−	+	+
H_2S production on TSI	+	+	U	V	−	−	+	V	−	−	+	−	F	V	−	−	−	−	−	−	−	−	+	+	+	−
α−haemolysis	−	−	−	−	−	−	−	−	F	F	−	+	F	−	−	V	−	−	−	+	−	−	V	V	+	U
Growth on:																										
2% (w/v) NaCl	+	+	−	−	−	−	U	+	−	F	+	−	V	−	−	−	−	+	V	F	−	U	−	−	−	−
1% (w/v) glycine	+	+	+	M	−	+	w	+	M	F	+	−	+	F	+	F	+	+	+	V	+	+	+	F	+	+
0.04% (w/v) TTC	w	U	V	+	U	V	U	−	+	−	−	V	+	+	−	−	+	U	−	−	+	−	F	−	−	+
mCCDA	−	+	U	+	−	U	−	+	+	F	U	M	M	+	+	+	+	+	V	+	U	U	+	+	+	U
Resistance to:																										
Nalidixic acid (30 mg L^{-1})	R	R	S	S	S	S	S	R	S	V	R	V	R	V	R	V	R	R	V	S	V	V	R	R	R	R
Cephalothin (30 mg L^{-1})	R	R	R	R	R	V	S	R	R	S	S	V	S	R	S	S	R	S	S	S	R	S	V	S	S	R

Positive: + (95-100%); M (70–95%); V (30–70%); F (10–30%); − (0-10%); w: weak growth/reaction; U: unknown/not determined
Resistance: S, R and V indicate sensitive, resistant and variable, respectively. §, urease-positive thermophilic campylobacters (UPTC)

Data are derived from the original species descriptions and/or Boukerb et al., *Int J Syst Evol Microbiol* 2019;69:3969-3979, On et al., *Int J Syst Evol Microbiol* 2017;67:5296-5311, Parisi et al., *Syst Appl Microbiol* 2021;44:126204 or Miller et al., *Int J Syst Evol Microbiol* 2024;74:006405

	jejuni doylei	*jejuni jejuni*	*lanienae*	*lari concheus*	*lari lari*	*magnus*	*majalis*	*massiliensis*	*mucosalis*	*novaezeelandiae*	*ornithocola*	*peloridis*	*pin. caledonicus*	*pin. pinnipediorum*	*portucalensis*	*rectus*	*showae*	*sput. fecalis*	*sput. paraureolyticus*	*sput. sputorum*	*subantarcticus*	*suis*	*taeniopygiae*	*upsaliensis*	*ureolyticus*	*volucris*	*vulpis*
Motility	+	+	+	+	+	+	U	−	+	+	+	+	+	+	−	+	+	+	+	+	+	U	+	+	−	+	+
Temperature (atmosphere)																											
37 °C (aerobic)	−	−	−	−	−	−	−	−	−	−	−	−	−	−	−	−	−	−	−	−	−	w	−	−	−	−	−
30 °C (microaerobic)	−	M	−	−	+	−	−	U	+	U	−	−	+	+	U	F	+	M	M	M	−	−	−	+	+	−	−
37 °C (microaerobic)	+	+	+	+	+	+	+	+	+	+	+	+	+	+	+	+	+	+	+	+	+	+	+	+	+	+	+
42 °C (microaerobic)	−	+	+	+	+	+	+	−	+	+	+	+	−	−	+	F	V	V	V	V	+	+	+	+	V	+	+
37 °C (anaerobic)	−	−	w	−	−	+	+	+	+	+	+	−	+	+	w	+	+	+	+	+	+	+	+	−	+	+	−
Oxidase	+	+	+	+	+	+	−	U	+	+	+	+	+	+	+	+	V	+	+	+	+	−	+	+	+	+	+
Catalase	M	+	+	+	+	+	−	U	−	+	+	+	−	+	−	F	V	+	−	−	+	−	+	−	F	+	−
Urease	−	−	−	−	V§	−	−	−	−	−	+	−	+	+	−	−	−	−	+	−	−	−	−	−	+	−	−
Alkaline phosphatase	−	−	+	U	−	V	U	−	M	−	−	−	U	U	U	−	−	−	−	−	U	U	−	−	−	−	V
Hippuricase	+	+	−	−	−	−	−	−	−	−	−	−	−	−	−	−	−	−	−	−	−	−	+	−	−	−	−
Indoxyl acetate hydrolysis	+	M	−	U	F	+	−	U	−	+	−	−	−	−	−	+	V	−	−	−	−	−	−	+	F	−	+
Reduction:																											
Nitrate	−	+	+	+	+	+	−	−	F	+	V	+	+	+	−	+	+	M	+	+	+	−	V	+	+	+	+
Selenite	−	M	V	U	V	+	U	U	F	−	U	U	U	U	U	−	−	V	V	V	−	U	+	+	−	+	+
TTC	V	M	+	U	M	−	U	−	−	F	F	−	U	U	U	−	−	−	−	−	U	U	+	V	−	−	−
H_2S production on TSI	−	−	−	U	−	−	+	−	+	−	−	U	+	+	−	−	V	+	+	+	−	−	−	−	−	−	−
α−haemolysis	+	+	+	U	+	−	U	U	−	+	−	U	+	+	−	+	+	+	+	+	+	U	−	+	V	U	+
Growth on:																											
2% (w/v) NaCl	−	−	−	+	M	−	−	U	M	+	U	M	U	U	−	V	+	+	+	+	+	−	−	−	+	−	−
1% (w/v) glycine	F	M	−	+	+	−	−	U	V	+	+	+	−	V	V	+	V	+	+	+	M	−	−	+	+	−	+
0.04% (w/v) TTC	V	M	V	U	M	+	−	U	−	F	U	U	U	U	−	−	−	−	−	−	U	−	+	V	−	−	U
mCCDA	+	+	+	+	+	+	+	U	+	U	U	+	−	−	U	−	+	M	M	M	U	+	+	+	V	U	+
Resistance to:																											
Nalidixic acid (30 mg L^{-1})	S	S	R	S	V	R	U	S	V	S	U	V	S	S	U	V	S	V	V	V	R	U	V	S	S	R	S
Cephalothin (30 mg L^{-1})	S	V	R	R	R	R	U	U	V	R	U	V	S	S	U	S	S	S	S	S	S	U	R	V	S	R	S

Positive: + (95-100%); M (70–95%); V (30–70%); F (10–30%); − (0-10%); w: weak growth/reaction; U: unknown/not determined

Re Resistance: S, R and V indicate sensitive, resistant and variable, respectively. §, urease-positive thermophilic campylobacters (UPTC)

Data are derived from the original species descriptions and/or Boukerb et al., *Int J Syst Evol Microbiol* 2019;69:3969-3979, On et al., *Int J Syst Evol Microbiol* 2017;67:5296-5311, Parisi et al., *Syst Appl Microbiol* 2021;44:126204 or Miller et al., *Int J Syst Evol Microbiol* 2024;74:006405
